# Riluzole reduces amyloid beta pathology, improves memory, and restores gene expression changes in a transgenic mouse model of early-onset Alzheimer’s disease

**DOI:** 10.1038/s41398-018-0201-z

**Published:** 2018-08-14

**Authors:** Masahiro Okamoto, Jason D. Gray, Chloe S. Larson, Syed Faraz Kazim, Hideaki Soya, Bruce S. McEwen, Ana C. Pereira

**Affiliations:** 1 Laboratory of Neuroendocrinology, The Rockefeller University, New York, NY 10065 USA; 2 Laboratory of Exercise Biochemistry and Neuroendocrinology, Faculty of Health and Sports Sciences, University of Tsukuba, Tsukuba, Ibaraki 305-8574 Japan; 3 Department of Neurology, Icahn School of Medicine at Mount Sinai, New York, NY 10029 USA; 4 Fishberg Department of Neuroscience, Friedman Brain Institute, Icahn School of Medicine at Mount Sinai, New York, NY 10029 USA

## Abstract

Alzheimer’s disease (AD) represents a major healthcare burden with no effective treatment. The glutamate modulator, riluzole, was shown to reverse many AD-related gene expression changes and improve cognition in aged rats. However, riluzole’s effect on amyloid beta (Aβ) pathology, a major histopathological hallmark of AD, remains unclear. 5XFAD transgenic mice, which harbor amyloid β precursor protein (APP) and presenilin mutations and exhibit early Aβ accumulation, were treated with riluzole from 1 to 6 months of age. Riluzole significantly enhanced cognition and reduced Aβ42, Aβ40, Aβ oligomers levels, and Aβ plaque load in 5XFAD mice. RNA-Sequencing showed that riluzole reversed many gene expression changes observed in the hippocampus of 5XFAD mice, predominantly in expression of canonical gene markers for microglia, specifically disease-associated microglia (DAM), as well as neurons and astrocytes. Central to the cognitive improvements observed, riluzole reversed alterations in NMDA receptor subunits gene expression, which are essential for learning and memory. These data demonstrate that riluzole exerts a disease modifying effect in an Aβ mouse model of early-onset familial AD.

## Introduction

Alzheimer’s disease (AD) is the most common neurodegenerative disorder, characterized by progressive memory loss and cognitive decline^1^. Histopathologically, AD exhibits an accumulation of amyloid plaques, formed of amyloid β (Aβ) peptide, and of neurofibrillary tangles made of abnormally hyperphosphorylated tau protein^2^. Presently, an estimated 5.5 million Americans suffer from AD, and the prevalence is expected to significantly increase in the coming decades as the population ages^3^. Currently, only symptomatic medications are available for AD and there are no pharmacological therapies that alter the progression of the disease. One of the greatest challenges for biomedical science today is to develop a disease-modifying therapy for AD and related neurodegenerative disorders.

Riluzole can prevent age-related cognitive decline in rats^4^ and in a transgenic mouse model of AD expressing mutant human tau^5^. Riluzole is FDA approved for the treatment of amyotrophic lateral sclerosis (ALS)^6^, and is known to modulate the glutamatergic system^5,7,8^. Clustering of dendritic spines in the hippocampus, which form the post-synaptic component of most excitatory synapses^9^, is one potential neuroplastic mechanism underlying riluzole’s efficacy^4^, because clustering of synaptic inputs has been shown to strengthen neural communication^10,11^. Additionally, riluzole was reported to rescue age-related gene expression changes in the rat hippocampus, along with many gene pathways implicated in AD^7^. The hippocampus is a region in the medial temporal lobe critical for learning and memory and one of the regions compromised first in the progression of AD, causing significant memory loss in affected individuals^12,13^.

However, the effect of riluzole on amyloid pathology, a hallmark of AD and considered to be one of the important triggers of the disease, is not well known. 5XFAD is a mouse model of early-onset AD that harbors five Aβ precursor protein (APP) and presenilin (PSEN) mutations linked to familial forms of AD (i.e., APP KM670/671NL (Swedish), APP I716V (Florida), APP V717I (London), PSEN1 M146L, and PSEN1 L286V) leading to a robust production of toxic Aβ peptides, amyloid plaque deposition, synaptic and neuronal loss, and memory deficits^14–16^. 5XFAD mice begin accumulating amyloid depositions as early as two months of age, with evidence of neuronal degeneration and synaptic loss starting at 4 months of age, which is coincident with significant memory impairment in 4–5-month-old 5XFAD mice^14^. The present study investigates riluzole’s effect on memory performance, Aβ pathology, and hippocampal gene expression profiles in 5XFAD transgenic mice. The results of this study provide further insight into the use of glutamate modulators as potential disease-modifying therapies for AD.

## Materials and methods

### Animals

Young male 5XFAD (tg6799) mice (Jackson Laboratories) and strain-matched C57BL/6 (wild type) mice (Jackson Laboratories) were housed in the animal vivarium at The Rockefeller University for the duration of the experiments. Mice were group-housed (3–5 animals/cage) in climate-controlled conditions (30–50% humidity, 21 ± 2 °C, 12:12 h light/dark cycle) with ad libitum access to food and water. Only male mice were used in this study to control for any possible sex differences in gene expression and behavior across the estrus cycle in female mice. All procedures were performed in accordance with approved protocols from The Rockefeller University’s Institutional Animal Care and Use Committee (IACUC), according to the PHS Policy on Humane Care and Use of Laboratory animals.

### Riluzole treatment

For these studies, one group of 5XFAD mice (*n* = 8) was given ad libitum access to riluzole solution, i.e., 13 mg/kg per mouse per day, a dose that has previously been reported to improve cognition and reduce tau pathology in P301L tau AD mice^5,17^, from 1 to 6 months of age (total treatment duration = 5 months). A control group of 5XFAD mice (*n* = 7) and the wild-type (WT) mice (*n* = 10) had ad libitum access to tap water. For immunohistochemical studies, a separate batch of 5XFAD mice was treated with riluzole (*n* = 5) or tap water as control (*n* = 5). We chose sample sizes based on past literature utilizing these methodologies^4,7,18–20^ with the intention to minimize the number of animals to be used for the present study.

Riluzole (R116; Sigma-Aldrich, St Louis, MO, USA) was dissolved in room temperature tap water at a stock concentration of 0.12 mg/ml and stirred for ~6 h, covered by foil to prevent light exposure. The stock solution was diluted into the mouse drinking water based on (1) average animal weight and, (2) water consumption over the previous 24 h period and throughout the study, which was measured by weighing the water bottles. We prepared fresh riluzole solution after every 2^nd^ to 3^rd^ day during the entire treatment period.

### Behavioral testing

The Y-maze was used to test hippocampal-dependent spatial memory retention as described previously^4^. One day before Y-maze testing, mice were habituated to an open field (OF) in the same room where the test would take place. Mice were acclimated to the room for 1 h before being placed in the OF (45 × 45 cm, ~20 lux on edges, ~40 lux in center) for 15 minutes. The OF was sprayed with 70% ethanol between trials. After 24 h, mice were tested in the Y-maze. During the 10-min acquisition phase, mice were placed in the “start” arm and allowed to explore the “start” and “familiar” arm while a “novel” arm was blocked. After a 1-hr delay, the mice were placed back in the “start” arm for a 10-min trial and allowed to explore all three arms. The dimensions of each arm were 9 cm × 40 cm. Corncob bedding from the cage of the mice being tested was mixed with clean bedding and placed evenly on the floor of the maze to reduce anxiety. The light in the maze was ~25 lux in the arms and ~40 lux in the center. To ensure that the preference for the novel arm was not based on external factors, the start, familiar, and novel arms were changed for each mouse. Cues of different sizes and patterns were placed on the curtain surrounding the maze facing each of the three arms to aid the mice in spatial orientation. Time in each arm, as well as frequency of entries, distance, and velocity were automatically recorded and tracked by Noldus Ethovision video tracking system and therefore were not blinded. Ratios of start / (start + familiar) for acquisition and novel / (novel + familiar) for trial were calculated using the time spent in those arms. The rationale for the test is that a mouse with intact memory should spend a higher percentage of time in the novel arm during the trial phase due to their tendency to explore novel environments.

### Tissue harvesting, immunoblotting, and ELISA

For biochemical studies, the mice were sacrificed by cervical dislocation, and brain tissue was immediately dissected into hippocampal and cortical regions, and flash frozen cortical brain tissue was homogenized in tissue homogenization buffer^21,22^ using a Teflon glass homogenizer. The BCA assay (23227, Thermo Fisher, IL, USA) was used to determine the protein concentrations of the homogenates. Six mice were selected at random from each group for the biochemical studies.

Western blotting was used to quantify the full-length APP (murine plus human) expression. 20 μg protein from each sample was resolved under reducing conditions on a 15-well, 4–12% Bis-Tris gel (Invitrogen, Carlsbad, CA) at 100 V for 90 min, then transferred to a PVDF membrane at 30 V for 90 min. The membrane was blocked in 1% non-fat powdered milk. An anti-APP/Aβ antibody (800704, Biolegend; reactive to amino acids 17–24 of APP/Aβ, recognizes both murine and human APP/Aβ; 4G8) was used at a 1:1,000 dilution; secondary antibody was anti-mouse at a 1:5,000 dilution. An anti-β-actin antibody (4970, Cell Signaling Technology, MA, USA) was used as a loading control at a 1:5,000 dilution; secondary antibody was anti-rabbit (R1006, Kindle Biosciences) at a 1:20,000 dilution. The membrane was imaged using KwikQuant digital imager (Kindle Biosciences).

Enzyme-linked immunosorbent assays (ELISAs) were used to quantify the amount of Aβ42 and Aβ40 peptide fragments and Aβ oligomers. Diethylamine (DEA)-soluble Aβ was extracted from the cortical brain tissue homogenate according to Casali and Landreth protocol^21,22^. The DEA-soluble portion was used in two ELISA kits (Aβ42: KMB3441, Invitrogen; Aβ40: KMB3481, Invitrogen) as per manufacturer’s instructions. Samples were diluted 1:2 for the Aβ40 ELISA and 1:10 for the Aβ42 ELISA. For Aβ oligomers, cortical brain tissue homogenate was diluted 1:10 and used in an ELISA kit (27725, IBL America) according to the manufacturer’s instructions. All ELISAs were read on a microplate reader (SpectraMax190; Molecular Devices) at 450 nm.

### Tissue processing and immunohistochemistry

For immunohistochemical studies, the mice were anesthetized with 125 mg/kg body weight of sodium pentobarbital intraperitoneally and transcardially perfused with 0.01 M phosphate buffered saline (PBS), followed by 4% paraformaldehyde (PFA) in 0.01 M PBS. After perfusion, the brains were removed from the skull immediately, and immersed in 4% PFA in 0.01 M PBS for 24–48 h, and then transferred to a 30% (w/v) sucrose solution at 4 °C for 24–48 h. The fixed brain tissues were stored at −80 °C till further analysis. Later, sagittal sections were cut at a thickness of 40 microns on a cryostat. The sections were stored in glycol anti-freeze solution (30% ethylene glycol and 30% glycerol in 0.01 M PBS) at −20 °C.

For TS + plaque load quantification, every 10^th^ section was chosen based on systematic random sampling (roughly 4–5 sections/animal) from 5 mice/group. A modified thioflavin-S staining protocol^23^ was used on free-floating brain sections, as reported previously^20,24^. Briefly, the sections were washed in copious volumes of distilled water and were then incubated in 0.25% KMnO_4_ for 4–5 min. After a brief wash with water, the sections were then treated with a solution of 1% K_2_S_2_O_5_ and 1% oxalic acid for 40–60 s until the brown color completely disappeared. Sections were then incubated in 0.05% thioflavin-S solution in dark for 8 min followed by two 1 min washes in 80% ethanol. Subsequently, the sections were washed thrice in distilled water for 1 min each. Sections were mounted and cover-slipped with Fluorogel mounting medium (Electron Microscopy Sciences, PA, USA). Maximum intensity projection images of confocal z-stacks were obtained with Leica SP5 DMI confocal microscope. Images were thresholded and TS^+^ plaque area in the subiculum region of the hippocampal formation and the frontal cortex was calculated using NIH Image J (v.1.46r).

### RNA-sequencing

Whole hippocampal mRNA was extracted from flash frozen tissue using the RNeasy Lipid Tissue Mini Kit (Qiagen; #74804). RNA concentration and quality were determined using a Bioanalyzer (Agilent). Samples with a RIN > 8 (RNA Integrity Number) were used for sequencing. Individual mice were separately pooled to generate three replicate sequencing libraries for each condition. The three libraries/group were prepared from the following mice: WT, *n* = 10 (pooled 3, 3, 4 mice); 5XFAD, *n* = 7 (pooled 2, 2, 3 mice); and 5XFAD-Riluzole, *n* = 8 (pooled 2, 3, 3 mice). Sequencing libraries were prepared using the TrueSeq Stranded mRNA Kit (Illumina) by the RU Genomics Core facility. Libraries were barcoded to allow for multiplexing in a single flow cell. 75 bp single strand reads were collected on a NexSeq 500 (Illumina) at a sequencing depth of ~45 million reads per sample.

Using Galaxy^25,26^ Fastq files were checked for quality by FastQC. Sequencing artifacts and residual adapter sequences were removed by trimming reads by 10 bp at the 5′ ends and then filtering to exclude reads with quality scores < 20. TopHat2^27^ was used to align read to the mouse genome (mm10). Bam files were loaded into Strand NGS (Agilent) for quantification of read density using DESeq. Significant genes in the differential expression analyses were identified using *z*-tests that were Benjamin–Hochberg corrected for false discovery rates at *p* < 0.05 and applying a cutoff of 1.5 × fold change. Venn diagrams, heatmaps, and scatter plots were generated using Strand NGS visualization tools.

Significant gene lists were uploaded to the DAVID bioinformatics website (http://david.abcc.ncifcrf.gov). Using the functional annotation clustering tool, enrichment scores, and GO terms were obtained. Enrichment scores above 1.3 were considered significant^28^. The enrichment scores from clusters with similar GO terms were used to compare pathways that were altered in both riluzole and 5XFAD conditions. Histograms were generated in Excel (Microsoft).

### qRT-PCR

Human APP transgene expression was determined using qRT-PCR. The cDNA was synthesized from 1500 ng of hippocampal mRNA using High Capacity cDNA Reverse Transcription Kit (Thermo Fisher Scientific). qRT-PCR was performed with QuantStudio 12 K Flex Real-Time PCR System (Thermo Fisher Scientific) using TaqMan probes (APP, Hs 00169098_m1) to detect the human APP transgene. Samples were run in triplicate with a 20 µl reaction volume and compared using the ∆∆CT method of relative quantification^29^. GAPDH (Mm 99999915_g1) was used as a normalization control.

### Data analysis

The details of RNA-Seq data analysis have been described in its relevant section. For behavioral and biochemical experiments, data were analyzed using one-way analysis of variance (ANOVA) followed by Tukey’s post hoc test. Pearson correlation analysis was used to study correlations between behavior and ELISA data. For immunohistochemistry data, the difference in Aβ plaque load between study groups was determined by employing Student’s *t*-test. The normality of the data was determined using the Shapiro–Wilk and Kolmogorov–Smirnov tests. There was no obvious difference in the variance between groups for each test. Statistical analysis was performed using GraphPad Prism 7.03 (GraphPad Software Inc., LaJolla, CA, USA). For all comparisons, *p* < 0.05 was considered as statistical significance level.

## Results

### Riluzole improves hippocampus-dependent memory in 5XFAD mice

**Fig. 1:**
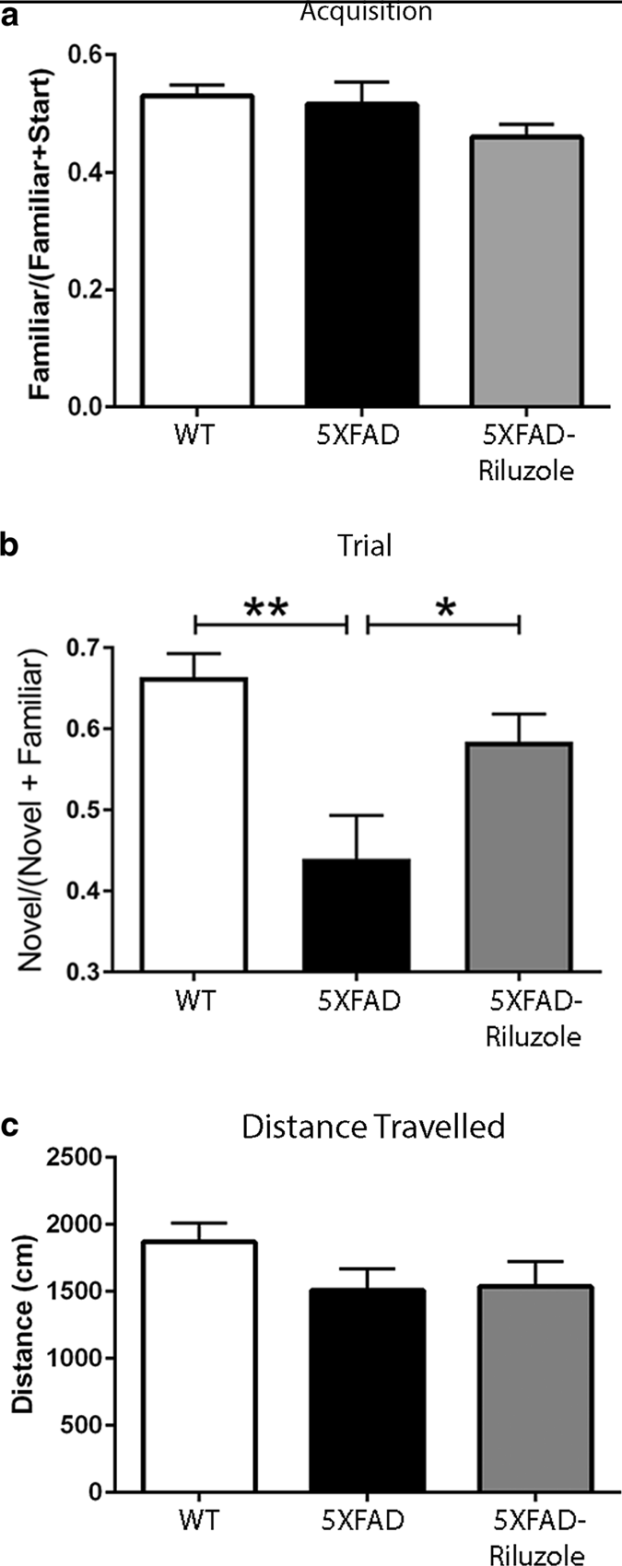
Riluzole treatment rescues cognitive impairment in 5XFAD mice. **a** In Y-Maze, there were no significant differences among groups in the ratio of time spent in familiar and start arms during the acquisition phase (*F*(2,22) = 2.269, *p* = 0.1271). **b** There were significant differences among groups in the ratio of time spent in the novel and familiar arms during the trial phase (*F*(2,21) = 8.736, *p* = 0.0017): between wild type (WT) and 5XFAD (*p* *=* 0.0012) and between 5XFAD and 5XFAD-Riluzole mice (*p* = 0.04), with WT and 5XFAD-Riluzole mice spending a significantly higher percentage of time in the novel arm. **c** There were no significant differences in distance traveled during the trial phase (*F*(2,21) = 1.673, *p* = 0.2117). The Y-maze data is presented as mean ± S.E.M., and based on WT, *n* = 10; 5XFAD, *n* = 6; and 5XFAD-Riluzole, *n* = 8 mice. *p *<* 0.05; **p < 0.01

5XFAD mice were treated with riluzole (13 mg/kg p.o.) from 1 month of age, prior to any evidence of amyloid accumulation in the 5XFAD mice, until 6 months of age, when the behavioral effects of amyloid accumulation are readily apparent^14^. Treated (5XFAD-Riluzole) and untreated (5XFAD) 5XFAD mice were compared against wild-type (WT) mice in the Y-maze, a hippocampus-dependent cognitive test^30,31^ at 4 and 6 months of age. No significant difference in Y-maze performance was observed at 4 months of age (data not shown), suggesting that 5XFAD mice had not yet manifested impaired memory. At 6 months of age, the acquisition trial did not show any difference amongst the three groups (Fig. 1a; *F*(2,22) = 2.269, *p* = 0.1271, one-way ANOVA). However, one animal in the 5XFAD group failed to show equal time in each arm during acquisition and therefore it was excluded from the final behavioral analysis (WT, *n* = 10; 5XFAD, *n* = 6; 5XFAD-Riluzole, *n* = 8). In the retention trial the percentage of time spent exploring the novel arm (exploration time in novel arm/exploration time in novel + familiar arm) showed a significant difference between the three groups (Fig. 1b; *F*(2,21) = 8.736, *p* = 0.0017, one-way ANOVA). 5XFAD mice showed significant memory impairment as compared to WT controls (Fig. 1b; *p* = 0.0012, Tukey’s post hoc test); riluzole treatment significantly ameliorated this deficit in 5XFAD mice (Fig. 1b; 5XFAD vs. 5XFAD-Riluolze, *p* = 0.04, Tukey’s post hoc test). Also, 5XFAD-Riluzole mice displayed similar performance in retention trial as WT controls, suggesting that the treatment with riluzole restored spatial reference memory of 5XFAD mice to WT control levels (Fig. 1b; *p* *=* 0.2615, Tukey’s post hoc test). The total distance traveled did not differ significantly among groups (Fig. 1c; *F*(2,21) = 1.673, *p* = 0.2117, one-way ANOVA), indicating that locomotor activity did not affect the results.

### Riluzole treatment reduces Aβ pathology that inversely correlates with memory performance in 5XFAD mice

**Fig. 2:**
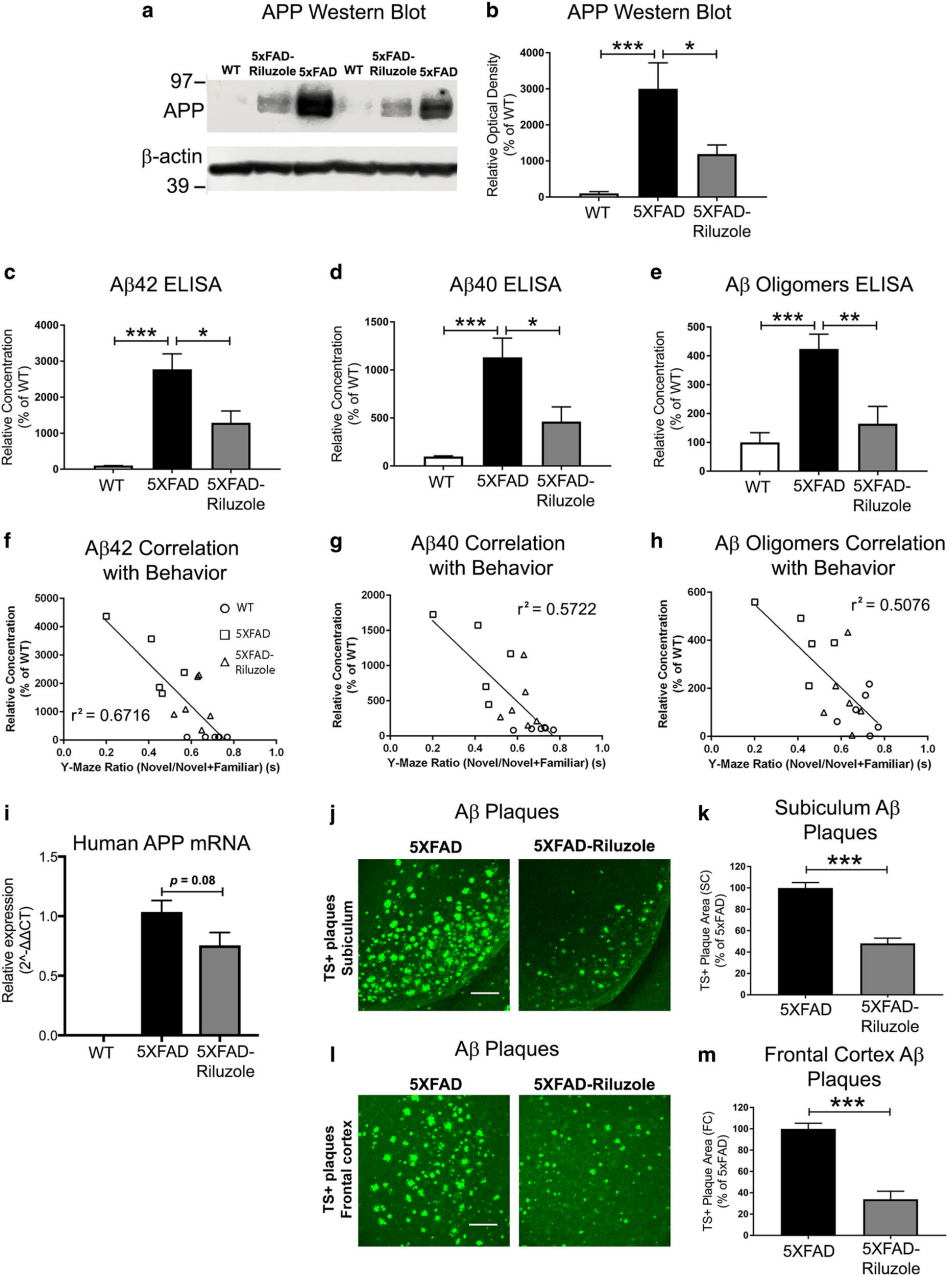
Riluzole treatment reduces Aβ pathology which inversely correlates with memory performance in 5XFAD mice. **a** Representative western blots of full-length APP protein expression are shown. The endogenous APP protein bands in WT mice appear faint because APP transgenic mice have significant overexpression, which required a short exposure time to obtain bands suitable for quantification of 5XFAD lanes. **b** Quantification data showing that APP protein expression was significantly different among groups (*F*(2,15) = 11.04, *p* *=* 0.0011): between wild type and 5XFAD (*p* = 0.0009) and between 5XFAD and 5XFAD-Riluzole mice (*p* = 0.0279). **c** Soluble Aβ42 was significantly different among groups (*F*(2,15) = 18.60, *p* < 0.0001): between wild type and 5XFAD (*p* *<* 0.001) and between 5XFAD and 5XFAD-Riluzole mice (*p* = 0.0108). **d** Soluble Aβ40 was significantly different among groups (*F*(2,15) = 12.90, *p* = 0.0005): between wild type and 5XFAD (*p* = 0.0004) and between 5XFAD and 5XFAD-Riluzole mice (*p* = 0.0141). **e** Aβ oligomers were significantly different among groups (*F*(2,15) = 12.00, *p* = 0.0008): between wild type and 5XFAD (*p* = 0.0009) and between 5XFAD and 5XFAD-Riluzole mice (*p* = 0.0057). **f** Aβ42 peptide (*r*^2^ = 0.6716, *p* < 0.0001), **g** Aβ40 peptide (*r*^2^ = 0.5722, *p* = 0.0004), and **h** Aβ oligomers (*r*^2^ = 0.5076, *p* = 0.0013) each showed a significant correlation between Aβ levels and memory performance in Y-maze. **i** The qRT-PCR analysis revealed no expression of human APP in WT mice, and no statistically significant difference between 5XFAD and 5XFAD-Riluzole (*p* = 0.08). **j**, **k** Riluzole treatment significantly reduced TS^+^ Aβ plaque area in the subiculum region of the hippocampus in 5XFAD mice (*p* < 0.001). **l**, **m** The Aβ plaque load in the frontal cortex was also significantly reduced by riluzole in 5XFAD mice (*p* < 0.001). The data in panels (**b**–**e**, **l**, **k**, **m**) is shown as mean ± S.E.M. The western blot data in panels (**a**, **b**) and ELISA data in panels (**c**–**e**) is based on WT, *n* = 6, 5XFAD, *n* = 6, and 5XFAD-Riluzole, *n* = 6. The correlation data in panels (**f**–**h**) is based on WT, *n* = 6, 5XFAD, *n* = 5, and 5XFAD, *n* = 6. The qRT-PCR data in panel (**l**) is based on WT, *n* = 10, 5XFAD, *n* = 7, 5XFAD-Riluzole, *n* = 8. The TS^+^ Aβ plaque load data in panels (**j**–**m**) is based on 5XFAD, *n* = 5, and 5XFAD-Riluzole, *n* = 5. *p *<* 0.05, **p *<* 0.01, ***p *<* 0.001. Scale bar = 100 µm

We utilized multiple methodologies to assess Aβ pathology including immunoblotting for full-length APP estimation, ELISAs for major Aβ toxic isoforms, Aβ 42 and Aβ 40, and Aβ oligomers, qRT-PCR based quantification of human APP mRNA transcripts, and immunohistochemical quantification of amyloid plaque load by thioflavin-S staining, to identify riluzole’s effect on different Aβ isoforms and aggregates that have been shown to have diverse roles in AD pathophysiology^32,33^. First, immunoblotting revealed a several-fold higher full-length APP protein expression in 5XFAD mice as compared to WT controls (Fig. 2a, b; *p* = 0.0009, Tukey’s post hoc test). Riluzole treatment significantly reduced APP protein expression in 5XFAD mice (Fig. 2a, b; *p* *=* 0.028, Tukey’s post hoc test). The APP protein expression did not differ significantly between WT and 5XFAD-Riluzole groups (*p* *=* 0.22; Tukey’s post hoc test). Second, an ELISA-based assay for the most toxic and amyloidogenic Aβ isoform, Aβ42, showed a significant difference between groups (Fig. 2c; *F*(2,15) = 18.60, *p* < 0.0001), with Tukey’s post hoc analysis showing a difference between WT and 5XFAD (*p* < 0.0001) and between 5XFAD and 5XFAD-Riluzole mice (*p* = 0.0108). Even though riluzole treatment significantly reduced Aβ42 levels, the levels were still significantly higher in 5XFAD-Riluzole mice as compared to WT controls (*p* *=* 0.0406). Third, Aβ40 ELISA showed a significant difference between groups (Fig. 2d; *F*(2,15) = 12.9, *p* = 0.0005), with Tukey’s post hoc analysis showing a statistically significant difference between WT and 5XFAD (*p* *=* 0.0004) and between 5XFAD and 5XFAD-Riluzole mice (*p* *=* 0.0141). No significant difference was found between 5XFAD-Riluzole and WT groups (*p* = 0.2166). Fourth, an ELISA-based assay for Aβ oligomers showed a significant difference between groups (Fig. 2e; *F*(2,15) = 12.00, *p* = 0.0008), with post hoc analysis showing a significant difference between WT and 5XFAD (*p* = 0.0009) and between 5XFAD and 5XFAD-Riluzole mice (*p* = 0.0057). There was no statistically significant difference between 5XFAD-Riluzole and WT groups (*p* = 0.6316). These results demonstrate that riluzole reduced the levels of toxic Aβ species, i.e., Aβ42, Aβ40, and Aβ oligomers.

Consistent with previous findings in 5XFAD and other APP/PSEN mutations AD mice^34,35^, levels of toxic Aβ species showed significant negative Pearson correlations with Y-maze performance with each of the isoforms tested, including Aβ42 (Fig. 2f; *r*^2^ = 0.6716, *p* < 0.0001), Aβ40 (Fig. 2g; *r*^2^ = 0.5722, *p* = 0.0004), Aβ oligomers (Fig. 2h; *r*^2^ = 0.5076, *p* = 0.0013). Notably, the strongest inverse correlation was with Aβ42, considered to be the most toxic Aβ species.

To determine if riluzole treatment altered APP transgene expression in 5XFAD mice, qRT-PCR using human APP specific primers was performed on hippocampal mRNA. The qRT-PCR analysis revealed, as expected, no expression of human APP in WT mice (Fig. 2i). The comparison of 5XFAD-Riluzole and 5XFAD controls revealed a trend towards reduction in riluzole-treated 5xFAD mice, however it did not reach statistical significance (Fig. 2i; *p* = 0.08, Student’s *t-*test).

We further evaluated the effect of riluzole treatment on Aβ pathology in 5XFAD mice by performing TS^+^-Aβ plaque load quantification (Fig. 2j–m). Quantification of TS^+^ stained Aβ plaques showed a significant reduction in riluzole-treated 5XFAD mice in the subiculum region of the hippocampus (Fig. 2j, k; 5XFAD vs. 5XFAD-Riluzole, Student’s *t*-test, *p* < 0.001). Also, riluzole treatment significantly reduced TS^+^-plaque area in the frontal cortex region of 5XFAD mice (Fig. 2l, m; 5XFAD vs. 5XFAD-Riluzole, Student’s *t*-test, *p* < 0.001).

### Riluzole treatment rescues differentially expressed genes in the hippocampus of 5XFAD mice

**Fig. 3:**
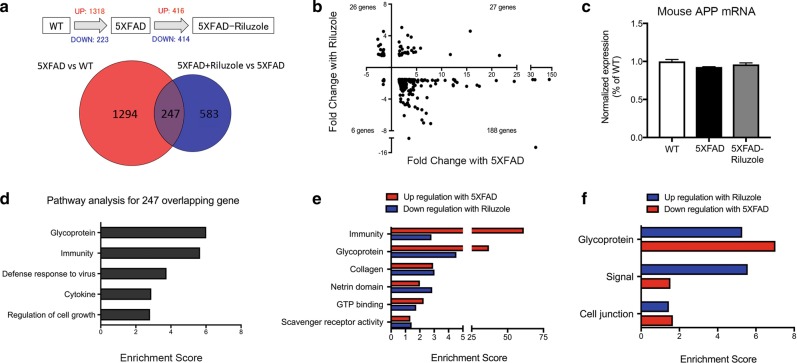
Treatment of 5XFAD mice with riluzole rescues gene expression changes in the hippocampus. **a** Venn diagram illustrating the overlap of 247 genes that were changed in 5XFAD compared to wild type and in 5XFAD mice treated with riluzole compare to untreated mice. **b** Scatter plot illustrating the 247 overlapping genes showing fold change by 5XFAD (*x*-axis) against fold change with riluzole treatment on 5XFAD mice (*y*-axis). 86% of overlapping 5XFAD genes is reversed by riluzole treatment. The upper left quadrant represents 26 genes that had decreased expression with 5XFAD and increased expression after riluzole treatment. Conversely, the lower-right quadrant illustrates 189 genes that were increased with 5XFAD and decreased by riluzole. **c** The mRNA expression of Mouse APP from RNA-Seq data revealed no significant difference among WT, 5XFAD, and 5XFAD-Riluzole groups. **d** Histograms illustrating significantly enriched pathways based on genes differentially expressed by overlapping gene. (enrichment score > 1.3). **e**, **f** Histograms illustrating significantly enriched pathways with the highest sum enrichment scores across opposite conditions (Increased in 5XFAD and Decreased with riluzole treatment or vice-versa) based on differentially expressed genes (enrichment score > 1.3). Similar pathways and enrichment scores were observed when comparing genes decreased by 5XFAD and increased by riluzole, as well as for genes increased with age and decreased by riluzole. The RNA-Seq data is based on hippocampal tissue pooled into three replicate sequencing libraries/group from WT, *n* = 10 (pooled 3, 3, 4 mice); 5XFAD, *n* = 7 (pooled 2, 2, 3 mice); and 5XFAD-Riluzole, *n* = 8 (pooled 2, 3, 3 mice)

Differential expression analysis of RNA-Sequencing (RNA-Seq) data from hippocampal mRNA from each condition revealed that untreated 5XFAD mice had significantly altered expression of 1541 genes (1318 upregulated and 223 downregulated) compared to WT mice (Fig. 3a). Riluzole treatment of 5XFAD mice altered expression of 830 (416 upregulated, 414 downregulated) genes compared to untreated 5XFAD mice (Fig. 3a). Importantly, 247 genes were found to be changed by both the 5XFAD transgene and riluzole treatment of the transgenic mice, suggesting they might be central not only to the pathology of AD, but also riluzole’s ability to reverse its effects. To further characterize this effect, a scatter plot of the fold change of the overlapping genes in each condition was generated (Fig. 3b). This showed riluzole treatment reversed the direction of change in expression for 86.6% of these genes, with 188 genes that increased with 5XFAD and were decreased with riluzole treatment (lower-right quadrant) and 26 genes that were decreased with 5XFAD and were increased with riluzole treatment (upper left quadrant). To investigate the effect of riluzole treatment on the endogenous APP, the expression of mouse APP mRNA was calculated from RNA-Seq data showing no significant difference in endogenous APP mRNA levels (Fig. 3c; *F*(2,6) = 0.6825, *p* = 0.09).

The overlapping 247 differentially expressed genes from the 5XFAD vs. WT and 5XFAD vs. 5XFAD-Riluzole comparisons were assigned to functional pathways using the DAVID bioinformatics database. Enrichment scores from common GO pathways were summed to identify groups of genes implicated in riluzole’s ability to rescue AD (Fig. 3d). Histograms of the summed enrichment scores illustrate pathways that were upregulated with 5XFAD and downregulated with riluzole, or downregulated with 5XFAD and upregulated with riluzole. Functional analysis via DAVID and assignment to GO categories revealed that riluzole reversed the pathways related to Immunity and Glycoproteins (Fig. 3e, f). For example, BMP4 and BMP6 are elevated in AD and implicated in neurogenesis^36,37^ and reversed by riluzole. Many of the immune genes were related to the innate immune system, such as IFIT3, CLEC7A, TRIM14, OAS2, and OAS3 (Table 1). The innate immune system has been implicated as a key mechanism of neuronal damage in AD^38^.

**Table 1:** Pathways and gene list that were reversed with riluzole treatment

Pathways	Genes that are upregulated with 5XFAD and downregulated with riluzole	Pathways	Genes that are downregulated with 5XFAD and upregulated with riluzole
Immunity	*BST2, CLEC7A, CYBB, H2-Q2, IFIT3, IRF7, LBP, LY9, MX1, MX2, Oas1a, OAS2, OAS3, Oasl2, RSAD2, TNFRSF17, TRIM14*	Glycoprotein	*Col6a4, HTR1B, IGFBPL1, IGSF9B, pcsk1, PI15, SLC9A3*
Glycoprotein	*1500015O10Rik, ABCA4, ACE, AQP1, BMP4, BMP6, BST2, Ccl9, CD5, CDH3, CLDN2, CLEC1A, CLEC7A, CLEC9A, COL17A1, COL4A3, COL4A4, COL8A1, COL8A2, COL9A3, CXCL5, CYBB, Defb11, ENPP2, F5, FAP, folr1, FOLR2, GLB1L2, H2-Q2, HCST, Ifi27l2a, IGFBP2, IGFBP5, IL15, ITPRIPL1, KCNE2, KL, KRT18, KRT8, LARGE2, LBP, LGALS3BP, LY9, MAMDC2, MFRP, Mfsd4b1, MIA, NOX1, oacyl, OAS2, OCA2, PRLR, SCTR, SEMA3B, SFRP1, SLAMF8, SLC2A12, SLC39A4, Slco1a5, SOSTDC1, SULF1, tmc8, TMPRSS11A, TNFRSF17, TNFSF8, TNS4, TTR, Vmn2r84, WFDC2, WFIKKN2*	Signal	*Col6a4, IGFBPL1, IGSF9B, pcsk1, PI15*
		Cell junction	*IGSF9B*
Collagen	*COL17A1, COL4A3, COL4A4, COL8A1, COL8A2, COL9A3, MFRP, TNFSF8*		
Netrin domain	*SFRP1, WFIKKN2*		
GTP binding	*9330111J21Rik1, GBP3, Gm12185, Gm4951, Gm5431, GNA15, MX1, MX2, RAB20, RHOD*		
Scanvenger receptor activity	*CD5, ENPP2, LGALS3BP*		

**Fig. 4:**
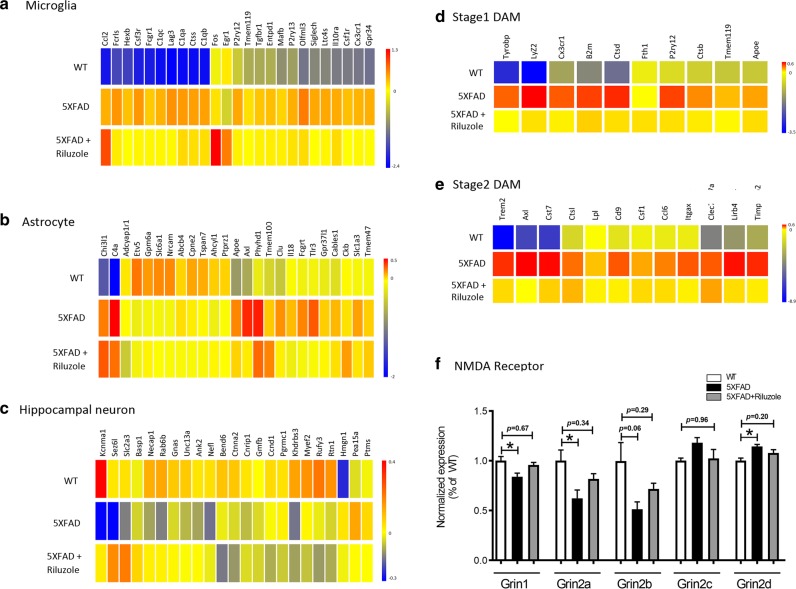
Expression changes in cell-type-specific markers and hippocampal NMDA receptor subunits are reversed by riluzole treatment. Heatmap showing 23–25 canonical expression markers for hippocampal **(a)** microglia^39^, **b** astrocytes^39^, **c** neurons^39^, and **(d**, **e)** a unique population of disease-associated microglia (DAM)^40^. Riluzole showed a rescue effect in neuronal and astrocytic populations. The rescue was most pronounced in microglia-related genes, and in particular DAM, which are associated with neurodegeneration. **f** 5XFAD mice have a significantly decreased expression levels of NMDA subunits Grin1 (*p* = 0.0417) and Grin2a (*p* = 0.0453), a trend towards reduced Grin2b (*p* = 0.065), and increased levels of Grin2d (*p* = 0.0251) in comparison to wild-type animals. Riluzole modulated the NMDA subunits expression levels in 5XFAD mice to WT control levels (5XFAD-Riluzole vs. WT; Grin1, *p* = 0.6764, Grin2a, *p* = 0.3420, Grin2b, *p* = 0.2969, Grin2c, *p* = 0.9620, Grin2d, *p* = 0.2059. The RNA-Seq data are based hippocampal tissue pooled into three replicate sequencing libraries/group from WT, *n* = 10 (pooled 3, 3, 4 mice); 5XFAD, *n* = 7 (pooled 2, 2, 3 mice); and 5XFAD-Riluzole, *n* = 8 (pooled 2, 3, 3 mice). *p < 0.05

Increasingly, researchers have characterized the distinct effects of AD on specific cell types of the brain. Genes that are canonically neuronal, astrocytic, or present primarily in microglia were selected based on a previous publication^39^ and heatmaps illustrating their change in expression across conditions were generated (Fig. 4a–c). Consistent with previous RNA-Seq studies in this mouse line^40,41^, microglia genes were nearly universally upregulated in 5XFAD mice, and many of them were downregulated by riluzole. For example, the microglia-related genes Clec7a and IRF7 (Table 1) expression are significantly downregulated in 5XFAD mouse hippocampus and rescued by riluzole. It has been previously reported that Aβ_42_ peptide induces IRF7 level and microglial inflammatory responses^42^. Thus, riluzole appears to exert an influence on several immune-related pathways implicated in AD. A recent publication characterizing expression changes in the 5XFAD mice identified a unique population of disease-associated microglia (DAM) as important for AD progression^40^. The present data confirm an upregulation of this specific microglia subclass of genes (DAM) in 5XFAD mice and that riluzole can reverse their upregulation (Fig. 4d, e).

A similar pattern was observed for many, but not all of the genes that are expressed predominately in neurons and astrocytes (Fig. 4b, c). CLU, a gene previously associated with AD by several GWAS studies, was rescued by riluzole^43,44^. Riluzole also reversed expression changes in genes related to neuroplasticity such, as ANK2, which is implicated in spectrin actin cytoskeleton^45^ and Nefl, important for neurofilaments^46^. Rescue also occurs in genes related to endocystosis, such as Necap1^47^, and those involved in the retromer transporter from endosome to Golgi, Rab6b^48^. Both endocytosis and the retromer have been implicated in AD pathophysiology^49,50^.

Proper expression and activation of hippocampal synaptic NMDA receptors is critical for long-term potentiation (LTP), learning, and memory^51,52^. In comparison to wild-type mice, 5XFAD mice were found to have significantly decreased expression levels of NMDA subunits Grin1 (Fig. 4f, *p* = 0.0417) and Grin2a (Fig. 4f, *p* = 0.0453), a trend towards reduced Grin2b expression (Fig. 4f, *p* = 0.065), and increased level of Grin2d (Fig. 4f, *p* = 0.0251). No statistically significant difference was observed in Grin2c expression between WT and 5XFAD mice (Fig. 4f, *p* = 0.18). There appeared to be a trend towards increase in Grin1, Grin2a, and Grin2b expression and a decrease in Grin2c and Grin2d expression in 5XFAD-Riluzole vs. 5XFAD mice (Fig. 4f). More importantly, the expression level of Grin1, Grin2a, Grin2b, Grin2c, and Grin2d did not differ significantly between 5XFAD-Riluzole vs. WT mice (Fig. 4f; *p* = 0.6764, *p* = 0.3420, *p* = 0.2969, p = 0.9620, and *p* = 0.2059, respectively). These data suggest that riluzole treatment normalized these NMDA subunits expression levels to WT controls.

## Discussion

This study showed that riluzole exerted a disease-modifying effect in an early-onset and aggressive mouse model of AD. It prevented hippocampus-dependent spatial memory decline in 5XFAD mice. A hallmark of the 5XFAD mouse model is elevated levels of Aβ. Riluzole treatment for 5 months significantly reduced full-length APP, Aβ42, Aβ40, Aβ oligomers levels, and Aβ plaque burden in the brains of 5XFAD mice. Levels of Aβ peptides strongly correlated with behavioral performance. Furthermore, riluzole reversed many of the gene expression changes resulting from the 5XFAD transgenes. The most significant effects were on immune pathways, and specifically microglia-related genes thought to be critical mediators of AD pathophysiology^53,54^. Riluzole treatment also reversed many of the changes in neuronal and astrocytic specific gene expression profiles.

In AD, toxic Aβ (mainly Aβ42 and Aβ40) deposition proceeds from oligomers to diffuse plaques, ultimately leading to the formation of compact plaques, which can be identified with thioflavin-S staining^20,23,24^. Even though the precise etiological mechanism responsible for neurodegeneration and cognitive impairment in AD remains elusive, evidence supports the hypothesis that Aβ triggers pathological cascades, which ultimately culminate in profound memory dysfunction^55–58^. Recent evidence further implicates Aβ-induced neural network dysfunction and hyperexcitability as a major contributor to cognitive impairment in AD^59,60^. In the present study, we found that riluzole significantly reduced Aβ pathology including toxic Aβ isoforms, oligomers, and the plaque load in 5XFAD mice. Remarkably, a strong inverse correlation was found between Aβ pathology and cognitive performance in these mice. This is in agreement with a previous study in 5XFAD mice, which concluded that it is ultimately cerebral amyloidosis that is responsible for neurodegeneration and cognitive impairment in these mice^61^. The present study suggests that the beneficial effect of riluzole on cognition in 5XFAD mice could be because of reduction in Aβ pathology.

The mechanism of Aβ reduction by riluzole treatment remains to be determined in future studies. In the present study, we found that riluzole treatment showed a trend towards reduction in human APP mRNA levels and a significant decrease in APP protein levels in 5XFAD mice. This hints towards a probable transcriptional downregulation and a likely translational downregulation of APP by riluzole. This could have contributed to a reduction in Aβ pathology in riluzole-treated 5XFAD mice, however, an effect of riluzole on amyloidogenic processing of APP or Aβ clearance cannot be ruled out. There also remains a possibility that at least some of the beneficial effects of riluzole could be because of APP reduction. APP was previously reported to be proinflammatory and to regulate microglial phenotype in APP/PS1 mice^62,63^. Also, APP overexpression was reported to be a key mediator of network hypersynchronous activity in AD mice^64^, recently hypothesized to be an important contributory factor to cognitive impairment in AD^60^. Thus, APP reduction by riluzole treatment in 5XFAD could have contributed to microglial downregulation and cognitive rescue. Nonetheless, as mentioned earlier, a previous study showed prevention of neurodegeneration and memory loss after genetic deletion of β-secretase (BACE1; major enzyme involved in amyloidogenic processing of APP) in 5XFAD mice^61^, suggesting the dominant role of Aβ in memory performance in these mice.

The identification of immune-related pathways is consistent with previous RNA-Seq results from 5XFAD mice^40,41^. Riluzole recovered several genes such as IFIT3, CLEC7A, TRIM14, OAS2, and OAS3 of the innate immune system, which have been thought to play a crucial role as disease promoting factors in AD^38^. A recent study performing single-cell transcriptional profiling in 5XFAD and wild-type mice revealed a microglia-like population associated with neurodegeneration^40^, and it identified activation genes as key mediators of disease pathology. Notably, riluzole reversed several of these genes including those related to TREM2 independent mechanisms, such as Cx3cr1, P2ry12, Tyrobp, Ctsb, B2m, and Lyz2, as well as TREM2 dependent genes, such as TREM2, Axl, Cst7, Ctsl, Ccl6, Itgax, Clec7a, and Lilrb4. These findings also converge with a growing human AD literature that suggest immune pathways, and most specifically the innate immune system and microglia-related genes as being importantly involved in the pathophysiology of AD^38,53,54^. Riluzole, therefore, rescues several immune-related pathways that may be critical in AD progression and cognitive dysfunction.

The effect of riluzole on cell-type-specific genes expressed predominantly in microglia, neurons, and astrocytes^39^ were investigated in 5XFAD mice. Heatmaps to visualize the expression levels of genes canonically associated with each of these cell types demonstrate that riluzole profoundly reverses the effects of the 5XFAD transgene on microglial gene expression, specifically the novel population of DAM^40^ (Fig. 4d, e). Further, expression levels of the gene CLU, which has been shown in multiple human genetic studies to be associated with an increased risk for AD^43,44^, could also be rescued in the 5XFAD mice treated with riluzole. Riluzole also rescued genes related to endocytosis (for example, Necap1)^47^ and the retromer transporter from endosome to Golgi, Rab6b^48^. Both endocytosis and the retromer dysfunction have been reported to be involved in the pathophysiology of AD in human genetic and neuropathologic studies^49,50^. Consistent with previous findings in aged rats, the identification of genes related to neural transmission and plasticity that were rescued by riluzole treatment^7^ support the hypothesis that clustering of dendritic spines is a potential mechanism by which riluzole is able to improve cognition^4^.

A growing scientific literature suggests glutamatergic dysregulation is an essential aspect of the pathophysiology of AD. First, excitatory pyramidal neurons are more vulnerable to cell death in AD^65,66^. The hippocampal and neocortical atrophy visible in AD brains demonstrates degeneration predominantly in large glutamatergic pyramidal neurons^66^. Second, β-amyloid and tau release and tau propagation are dependent on excitatory neural activity^67–70^. Third, toxicity of these AD-related proteins is likely dependent on glutamatergic dysfunction. For example, Aβ oligomers have been shown to disrupt glutamate transporters^71^, inhibiting LTP, critical for learning, and memory, and leading to activation of extrasynaptic NMDA receptors^72^, which has been associated with long-term depression and excitotoxicity^73^. We have observed in this study that riluzole rescued hippocampal expression of NMDA receptor subunits in 5XFAD to wild-type levels which play an essential role in LTP, learning, and memory formation^74,75^. These NMDA receptor subunits included synaptic NMDA receptor Grin2a (NMDAR2A, GluN2A) and the more abundant extrasynaptic NMDA receptor Grin2b (NMDAR2B, GluN2B)^76,77^, suggesting riluzole may affect the NMDA receptor subunits dynamics in a complex manner in both synaptic and extrasynaptic spatial domains. The timing and magnitude of activation of these receptors should be investigated in future studies as transient and intense activation of synaptic NMDA receptors by trans-synaptic glutamate release has been thought to be neuroprotective and important for learning and memory while a chronic activation of extrasynaptic NMDA receptors by sustained glutamate elevation in the extrasynaptic space could contribute to excitotoxicity^73^.

Importantly, activation and maturation of microglia is dependent on glutamate levels^78,79^, which could possibly explain the robust effect on microglia-related gene expression we observe with the glutamate modulator riluzole treatment. In addition, Aβ is known to induce microglia^80–82^ and the observed DAM reversal in treated animals could be an effect of reduced Aβ levels induced by riluzole or other potential mechanisms. In summary, glutamatergic dysregulation appears to be implicated in a cycle of toxicity in AD through several pathways, including microglia-mediated neuroinflammation, excitotoxicity, release and toxicity of Aβ, and tau release and tau propagation. We hypothesize that modulation of glutamatergic neurons and synapses in AD, the most susceptible to degeneration and the best predictor of cognitive decline in AD^83–85^, can significantly mitigate toxicities through multiple pathways in AD.

Future studies will investigate the mechanisms through which the glutamate modulator riluzole prevented cognitive decline in this aggressive amyloid pathology mouse model of AD. Together, these findings suggest that riluzole’s regulation of the glutamatergic synapse plays a critical role in reducing Aβ levels and restoring expression of genes implicated in both microglial activation and synaptic transmission.

## References

[1] McKhann G (2011). The diagnosis of dementia due to Alzheimer’s disease: recommendations from the National Institute on Aging- Alzheimer’s Association workgroups on diagnostic guidelines for Alzheimer’s disease. Alzheimers Dement..

[2] Braak H, Braak E (1996). Evolution of the neuropathology of Alzheimer’s disease. Acta Neurol. Scand..

[3] Alzheimer’s Association. (2017). 2017 Alzheimer’s Disease Facts and Figures. Alzheimers Dement..

[4] Pereira AC (2014). Glutamatergic regulation prevents hippocampal-dependent age-related cognitive decline through dendritic spine clustering. Proc. Natl Acad. Sci. USA.

[5] Hunsberger HC (2015). Riluzole rescues glutamate alterations, cognitive deficits, and tau pathology associated with P301L tau expression. J. Neurochem..

[6] Bensimon G, Lacomblez L, Meininger V (1994). A controlled trial of riluzole in amyotrophic lateral sclerosis. ALS/Riluzole Study Group. N. Engl. J. Med..

[7] Pereira AC (2017). Age and Alzheimer’s disease gene expression profiles reversed by the glutamate modulator riluzole. Mol. Psychiatry.

[8] Banasr M (2010). Glial pathology in an animal model of depression: reversal of stress-induced cellular, metabolic and behavioral deficits by the glutamate-modulating drug riluzole. Mol. Psychiatry.

[9] Trachtenberg JT (2002). Long-term in vivo imaging of experience-dependent synaptic plasticity in adult cortex. Nature.

[10] Larkum ME, Nevian T (2008). Synaptic clustering by dendritic signalling mechanisms. Curr. Opin. Neurobiol..

[11] Polsky A, Mel BW, Schiller J (2004). Computational subunits in thin dendrites of pyramidal cells. Nat. Neurosci..

[12] Fox NC (1996). Presymptomatic hippocampal atrophy in Alzheimer’s disease. A longitudinal MRI study. Brain.

[13] West MJ, Coleman PD, Flood DG, Troncoso JC (1994). Differences in the pattern of hippocampal neuronal loss in normal aging and Alzheimers-disease. Lancet.

[14] Oakley H (2006). Intraneuronal β-Amyloid aggregates, neurodegeneration, and neuron loss in transgenic mice with five familial Alzheimer’s disease mutations: potential factors in amyloid plaque formation. J. Neurosci..

[15] Kimura R, Ohno M (2009). Impairments in remote memory stabilization precede hippocampal synaptic and cognitive failures in 5XFAD Alzheimer mouse model. Neurobiol. Dis..

[16] Eimer WA, Vassar R (2013). Neuron loss in the 5XFAD mouse model of Alzheimer’s disease correlates with intraneuronal Aβ42 accumulation and Caspase-3 activation. Mol. Neurodegener..

[17] Gourley SL, Espitia JW, Sanacora G, Taylor JR (2012). Antidepressant-like properties of oral riluzole and utility of incentive disengagement models of depression in mice. Psychopharmacol. (Berl.).

[18] Colié S (2017). Neuronal p38α mediates synaptic and cognitive dysfunction in an Alzheimer’s mouse model by controlling β-amyloid production. Sci. Rep..

[19] Boza-Serrano A, Yang Y, Paulus A, Deierborg T (2018). Innate immune alterations are elicited in microglial cells before plaque deposition in the Alzheimer’s disease mouse model 5xFAD. Sci. Rep..

[20] Kazim SF (2014). Disease modifying effect of chronic oral treatment with a neurotrophic peptidergic compound in a triple transgenic mouse model of Alzheimer’s disease. Neurobiol. Dis..

[21] Gelaye B, Rondon M, Araya PR (2016). A PM. Aβ extraction from murine brain homogenates. Bio Protoc..

[22] Casali BT (2015). Omega-3 fatty acids augment the actions of nuclear receptor agonists in a mouse model of Alzheimer’s disease. J. Neurosci..

[23] Vallet PG (1992). A comparative study of histological and immunohistochemical methods for neurofibrillary tangles and senile plaques in Alzheimer’s disease. Acta Neuropathol..

[24] Dai C (2015). Passive immunization targeting the N-terminal projection domain of tau decreases tau pathology and improves cognition in a transgenic mouse model of Alzheimer disease and tauopathies. J. Neural Transm..

[25] Goecks J, Nekrutenko A, Taylor J, Galaxy Team T. (2010). Galaxy: a comprehensive approach for supporting accessible, reproducible, and transparent computational research in the life sciences. Genome Biol..

[26] Blankenberg D (2010). Galaxy: A Web-Based Genome Analysis Tool for Experimentalists. Current Protocols in Molecular Biology..

[27] Kim D (2013). TopHat2: accurate alignment of transcriptomes in the presence of insertions, deletions and gene fusions. Genome Biol..

[28] Huang DW, Sherman BT, Lempicki RA (2009). Systematic and integrative analysis of large gene lists using DAVID bioinformatics resources. Nat. Protoc..

[29] Livak KJ, Schmittgen TD (2001). Analysis of relative gene expression data using real-time quantitative PCR and the 2(-Delta Delta C(T)) Method. Methods.

[30] Conrad CD, Galea LA, Kuroda Y, McEwen BS (1996). Chronic stress impairs rat spatial memory on the Y maze, and this effect is blocked by tianeptine pretreatment. Behav. Neurosci..

[31] Dellu F, Mayo W, Cherkaoui J, Le Moal M, Simon H (1992). A two-trial memory task with automated recording: study in young and aged rats. Brain Res..

[32] Benilova I, Karran E, De Strooper B (2012). The toxic Aβ oligomer and Alzheimer’s disease: an emperor in need of clothes. Nat. Neurosci..

[33] Haass C, Selkoe DJ (2007). Soluble protein oligomers in neurodegeneration: lessons from the Alzheimer’s amyloid β-peptide. Nat. Rev. Mol. Cell Biol..

[34] Gordon MN (2001). Correlation between cognitive deficits and Abeta deposits in transgenic APP + PS1 mice. Neurobiol. Aging.

[35] Girard SD (2014). Onset of hippocampus-dependent memory impairments in 5XFAD transgenic mouse model of Alzheimer’s disease. Hippocampus.

[36] Crews L (2010). Increased BMP6 levels in the brains of Alzheimer’s disease patients and APP transgenic mice are accompanied by impaired neurogenesis. J. Neurosci..

[37] Li D (2008). Decreased hippocampal cell proliferation correlates with increased expression of BMP4 in the APPswe/PS1??E9 mouse model of Alzheimer’s disease. Hippocampus.

[38] Heneka MT, Golenbock DT, Latz E (2015). Innate immunity in Alzheimer’s disease. Nat. Immunol..

[39] Butovsky O (2014). Identification of a unique TGF-β-dependent molecular and functional signature in microglia. Nat. Neurosci..

[40] Keren-Shaul H (2017). A unique microglia type associated with restricting development of Alzheimer’s disease. Cell.

[41] Landel V (2014). Temporal gene profiling of the 5XFAD transgenic mouse model highlights the importance of microglial activation in Alzheimer’s disease. Mol. Neurodegener..

[42] Woodling NS (2014). Suppression of Alzheimer-associated inflammation by microglial prostaglandin-E2 EP4 receptor signaling. J. Neurosci..

[43] Lambert JC (2009). Genome-wide association study identifies variants at CLU and CR1 associated with Alzheimer’s disease. Nat. Genet..

[44] Harold D (2009). Genome-wide association study identifies variants at CLU and PICALM associated with Alzheimer’s disease. Nat. Genet..

[45] Goellner B, Aberle H (2012). The synaptic cytoskeleton in development and disease. Dev. Neurobiol..

[46] Fernandez-Martos CM, King AE, Atkinson RAK, Woodhouse A, Vickers JC (2015). Neurofilament light gene deletion exacerbates amyloid, dystrophic neurite, and synaptic pathology in the APP/PS1 transgenic model of Alzheimer’s disease. Neurobiol. Aging.

[47] Ritter B (2013). NECAP 1 regulates AP-2 interactions to control vesicle size, number, and cargo during clathrin-mediated endocytosis. PLoS Biol..

[48] Monier S, Jollivet F, Janoueix-Lerosey I, Johannes L, Goud B (2002). Characterization of novel Rab6-interacting proteins involved in endosome-to-TGN transport. Traffic.

[49] Small SA, Petsko GA (2015). Retromer in Alzheimer disease, Parkinson disease and other neurological disorders. Nat. Rev. Neurosci..

[50] Nixon Ra, Cataldo AM, Mathews PM (2000). The endosomal-lysosomal system of neurons in Alzheimer’s disease pathogenesis: a review. Neurochem. Res..

[51] Li F, Tsien JZ (2009). Memory and the NMDA receptors. N. Engl. J. Med..

[52] Collingridge GL, Bliss TVP (1987). NMDA receptors—their role in long-term potentiation. Trends Neurosci..

[53] Streit WJ (2004). Microglia and Alzheimer’s disease pathogenesis. J. Neurosci. Res..

[54] Colonna M, Wang Y (2016). TREM2 variants: new keys to decipher Alzheimer disease pathogenesis. Nat. Rev. Neurosci..

[55] Hardy J, Selkoe DJ (2002). The amyloid hypothesis of Alzheimer’s disease: progress and problems on the road to therapeutics. Science.

[56] Selkoe DJ, Schenk D (2003). Alzheimer’s disease: molecular understanding predicts amyloid-based therapeutics. Annu. Rev. Pharmacol. Toxicol..

[57] LaFerla FM, Oddo S (2005). Alzheimer’s disease: Abeta, tau and synaptic dysfunction. Trends Mol. Med..

[58] Mucke L, Selkoe DJ (2012). Neurotoxicity of amyloid β-protein: synaptic and network dysfunction. Cold Spring Harb. Perspect. Med..

[59] Palop JJ, Mucke L (2016). Network abnormalities and interneuron dysfunction in Alzheimer disease. Nat. Rev. Neurosci..

[60] Palop JJ, Mucke L (2010). Amyloid-beta-induced neuronal dysfunction in Alzheimer’s disease: from synapses toward neural networks. Nat. Neurosci..

[61] Ohno M (2007). BACE1 gene deletion prevents neuron loss and memory deficits in 5XFAD APP/PS1 transgenic mice. Neurobiol. Dis..

[62] Manocha GD (2016). APP regulates microglial phenotype in a mouse model of Alzheimer’s disease. J. Neurosci..

[63] Sondag CM, Combs CK (2004). Amyloid precursor protein mediates proinflammatory activation of monocytic lineage cells. J. Biol. Chem..

[64] Born HA (2014). Genetic suppression of transgenic APP rescues Hypersynchronous network activity in a mouse model of Alzeimer’s disease. J. Neurosci..

[65] Morrison JH, Hof PR (1997). Life and death of neurons in the aging brain. Science.

[66] Morrison JH, Hof PR (2002). Selective vulnerability of corticocortical and hippocampal circuits in aging and Alzheimer’s disease. Prog. Brain. Res..

[67] Kamenetz F (2003). APP processing and synaptic function. Neuron.

[68] Yamada K (2014). Neuronal activity regulates extracellular tau in vivo. J. Exp. Med..

[69] Pooler AM, Phillips EC, Lau DHW, Noble W, Hanger DP (2013). Physiological release of endogenous tau is stimulated by neuronal activity. EMBO Rep..

[70] Wu JW (2016). Neuronal activity enhances tau propagation and tau pathology in vivo. Nat. Neurosci..

[71] Li S (2009). Soluble oligomers of amyloid β protein facilitate hippocampal long-term depression by disrupting neuronal glutamate uptake. Neuron.

[72] Li S (2011). Soluble A oligomers inhibit long-term potentiation through a mechanism involving excessive activation of extrasynaptic NR2B-containing NMDA receptors. J. Neurosci..

[73] Hardingham GE, Bading H (2010). Synaptic versus extrasynaptic NMDA receptor signalling: implications for neurodegenerative disorders. Nat. Rev. Neurosci..

[74] Williams JM (2003). Long-term regulation of N-methyl-D-aspartate receptor subunits and associated synaptic proteins following hippocampal synaptic plasticity. Neuroscience.

[75] Tsien JZ, Huerta PT, Tonegawa S (1996). The essential role of hippocampal CA1 NMDA receptor-dependent synaptic plasticity in spatial memory. Cell.

[76] Martel MA, Wyllie DJA, Hardingham GE (2009). In developing hippocampal neurons, NR2B-containing N-methyl-d-aspartate receptors (NMDARs) can mediate signaling to neuronal survival and synaptic potentiation, as well as neuronal death. Neuroscience.

[77] Groc L (2006). NMDA receptor surface mobility depends on NR2A-2B subunits. Proc. Natl Acad. Sci. USA.

[78] Christensen RN, Ha BK, Sun F, Bresnahan JC, Beattie MS (2006). Kainate induces rapid redistribution of the actin cytoskeleton in ameboid microglia. J. Neurosci. Res..

[79] Liu GJ, Nagarajah R, Banati RB, Bennett MR (2009). Glutamate induces directed chemotaxis of microglia. Eur. J. Neurosci..

[80] Weldon DT (1998). Fibrillar beta-amyloid induces microglial phagocytosis, expression of inducible nitric oxide synthase, and loss of a select population of neurons in the rat CNS in vivo. J. Neurosci..

[81] Bamberger ME, Landreth GE (2001). Microglial interaction with beta-amyloid: implications for the pathogenesis of Alzheimer’s disease. Microsc. Res. Tech..

[82] Heurtaux T (2010). Microglial activation depends on beta-amyloid conformation: role of the formylpeptide receptor 2. J. Neurochem..

[83] DeKosky ST, Scheff SW (1990). Synapse loss in frontal cortex biopsies in Alzheimer’s disease: correlation with cognitive severity. Ann. Neurol..

[84] Selkoe DJ (2002). Alzheimer’s disease is a synaptic failure. Science.

[85] Terry RD (1991). Physical basis of cognitive alterations in Alzheimer’s disease: synapse loss is the major correlate of cognitive impairment. Ann. Neurol..

