# Correction: Riluzole reduces amyloid beta pathology, improves memory, and restores gene expression changes in a transgenic mouse model of early-onset Alzheimer’s disease

**DOI:** 10.1038/s41398-019-0408-7

**Published:** 2019-02-04

**Authors:** Masahiro Okamato, Jason D. Gray, Chloe S. Larson, Syed Faraz Kazim, Hideaki Soya, Bruce S. McEwen, Ana C. Pereira

**Affiliations:** 1 Laboratory of Neuroendocrinology, The Rockefeller University, New York, NY 10065 USA; 2 Laboratory of Exercise Biochemistry and Neuroendocrinology, Faculty of Health and Sports Sciences, University of Tsukuba, Tsukuba, Ibaraki 305-8574 Japan; 3 Department of Neurology, Icahn School of Medicine at Mount Sinai, New York, NY 10029 USA; 4 Fishberg Department of Neuroscience, Friedman Brain Institute, Icahn School of Medicine at Mount Sinai, New York, NY 10029 USA

**Correction to: Translational Psychiatry;**

10.1038/s41398-018-0201-z;

Published online 14 August 2018.

The author’s name was spelled incorrectly as “Masahir Okamoto”. This has been updated to “Masahiro Okamoto” in the HTML and PDF of the article.

